# Evaluation of Sleep Quality and Fatigue in Patients with Usher Syndrome Type 2a

**DOI:** 10.1016/j.xops.2023.100323

**Published:** 2023-05-05

**Authors:** Jessie M. Hendricks, Juriaan R. Metz, Hedwig M. Velde, Jack Weeda, Franca Hartgers, Suzanne Yzer, Carel B. Hoyng, Ronald J.E. Pennings, Rob W.J. Collin, Myrthe H.M. Boss, Erik de Vrieze, Erwin van Wijk

**Affiliations:** 1 Department of Otorhinolaryngology, Donders Institute for Brain, Cognition and Behaviour, Radboud University Medical Center, Nijmegen, The Netherlands; 2 Department of Animal Ecology & Physiology, Radboud Institute for Biological and Environmental Sciences, Radboud University Nijmegen, The Netherlands; 3 Department of Ophthalmology, Radboud University Medical Center, Nijmegen, The Netherlands; 4 Department of Human Genetics, Radboud University Medical Center, Nijmegen, The Netherlands; 5 Department of Neurology, Hospital Gelderse Vallei, Ede, The Netherlands

**Keywords:** Fatigue, Questionnaires, Sleep, USH2a, Usher syndrome

## Abstract

**Purpose:**

To study the prevalence, level, and nature of sleep problems and fatigue experienced by Usher syndrome type 2a (USH2a) patients.

**Design:**

Cross-sectional study.

**Participants:**

Fifty-six genetically confirmed Dutch patients with syndromic USH2a and 120 healthy controls.

**Methods:**

Sleep quality, prevalence, and type of sleep disorders, chronotype, fatigue, and daytime sleepiness were assessed using 5 questionnaires: (1) Pittsburgh Sleep Quality Index, (2) Holland Sleep Disorders Questionnaire, (3) Morningness–Eveningness Questionnaire, (4) Checklist Individual Strength, and (5) Epworth Sleepiness Scale. For a subset of patients, recent data on visual function were used to study the potential correlation between the outcomes of the questionnaires and disease progression.

**Main Outcome Measures:**

Results of all questionnaires were compared between USH2a and control cohorts, and the scores of the patients were compared with disease progression defined by age, visual field size, and visual acuity.

**Results:**

Compared with the control population, patients with USH2a experienced a poorer quality of sleep, a higher incidence of sleep disorders, and higher levels of fatigue and daytime sleepiness. Intriguingly, the sleep disturbances and high levels of fatigue were not correlated with the level of visual impairment. These results are in accordance with the patients’ experiences that their sleep problems already existed before the onset of vision loss.

**Conclusions:**

This study demonstrates a high prevalence of fatigue and poor sleep quality experienced by patients with USH2a. Recognition of sleep problems as a comorbidity of Usher syndrome would be a first step toward improved patient care. The absence of a relationship between the level of visual impairment and the severity of reported sleep problems is suggestive of an extraretinal origin of the sleep disturbances.

**Financial Disclosure(s):**

Proprietary or commercial disclosure may be found after the references.

Usher syndrome (USH) is an autosomal recessively inherited disorder, mainly affecting the sensory cells in the cochlea and retina, characterized by congenital sensorineural hearing impairment and progressive vision loss resulting from retinitis pigmentosa (RP). Usher syndrome is genetically and clinically heterogeneous. To date, 4 clinical types can be distinguished as follows: (1) USH type 1 (USH1), (2) USH type 2 (USH2), (3) USH type 3 (USH3), and (4) USH type 4 (USH4).^1^^,^^2^ USH1 is characterized by severe to profound congenital hearing loss, detection of the initial clinical symptoms of RP before puberty, and impaired vestibular function. Usher syndrome type 2 is the most common type. Symptoms of USH2 are moderate to severe congenital hearing impairment and RP presenting around puberty or during early adulthood. Usher syndrome type 3 and USH4 are both characterized by progressive hearing loss, and, in the case of USH4, it is late onset. The onset of RP is variable in USH3 patients, whereas USH4 is characterized by late-onset RP.^2^^,^^3^ USH is estimated to have a global prevalence of approximately 1 in 20 000—a total of approximately 400 000 patients worldwide—representing approximately 50% of all cases with a type of hereditary deaf-blindness.^1^^,^^4^ Approximately two-thirds of all Usher patients have USH2, of which up to 85% can be explained by pathogenic variants in the *USH2A* gene.^5^, ^6^, ^7^ Besides hearing impairment and vision loss, comorbidities like olfactory deficits and deficits in tactile perception have also been associated with USH.^8^, ^9^, ^10^, ^11^ In addition, patients often report fatigue and sleep problems that severely impact their quality of life. Although often mentioned during anamneses, little is known about the origin of these problems. So far, fatigue has not been recognized as a hallmark symptom of USH. Instead, it is often seen as a consequence of the double sensory disability.

However, it is well established that circadian disorders are frequently found in the visually impaired population, especially in individuals without residual light perception.^12^ Loss of light perception generally results in a loss of synchronization of the circadian clock, thus explaining the high prevalence of sleep disorders among the blind community.^13^ Although multiple studies have been performed on blindness-associated sleep disorders in general, little is known about the specific sleep disturbances experienced by USH patients. Only a few publications briefly report sleep deficiencies within the USH community. For example, Dammeyer^14^ studied the development and characteristics of children with USH. In his study, sleep problems were reported in children with USH that were still largely asymptomatic for vision impairment. Twenty of the 26 included individuals with USH experienced some degree of sleep problems. Dammeyer’s^14^ study shows the high prevalence of sleep disturbances in USH patients, as well as the finding that this comorbidity is already apparent in relatively well-sighted individuals. Despite the high prevalence, the nature of the reported sleep disturbances was not discussed in detail in this report. There was no information about the type of sleep disturbance, and the clinical type of USH was not reported in relation to the level of sleep disturbance. In addition to the study of Dammeyer,^14^ there are a few reports on the psychological health of USH1 and USH2 patients.^15^, ^16^, ^17^ In all these studies, fatigue is a recurrent topic. These studies, however, either did not focus on sleep at all,^16^^,^^17^ or the presence of sleep problems was only reported by one dichotomous question.^15^ To get a better understanding of fatigue and sleep problems in USH patients, a more extensive survey is needed. First, participants should have a conclusive genetic diagnosis of USH and, ideally, should also have available data on their current state of visual function.

In the present manuscript, 5 validated questionnaires were used to assess the presence, nature, and prevalence of sleep problems and fatigue in a cohort of USH type 2a (USH2a) patients. A total of 56 genetically confirmed USH2a patients were included, representing approximately 15% of the Dutch USH2a patient population. Recent data on visual function were available for a subset of the participants, which were used to investigate the potential correlation between the outcomes of the questionnaires and level of visual dysfunction. To the best of our knowledge, this is the first study to specifically investigate and quantify sleep disturbances and fatigue in USH2a patients. Recognizing this potential comorbidity of USH is the first step toward an improvement in personalized patient care, which will eventually benefit these patients’ quality of life.

## Methods

### Ethics, Inclusion, and Recruitment

The study adhered to the Declaration of Helsinki and was classified as non-WMO (Wet Medisch-wetenschappelijk Onderzoek [Medical Scientific Research Act]) research by the local medical ethics review committee. Therefore, no full review according to the Dutch Medical Research with Human Subjects Law was performed. For this study, syndromic USH2a patients with a conclusive genetic diagnosis and healthy controls who were aged ≥ 18 years were asked to participate. The level of remaining visual function was not an inclusion criterion. The survey was only spread among the Dutch USH2a population and administered within a short period of time to minimize differences in lifestyle and the amount of captured natural daylight and to prevent language-induced differences in question interpretation. The survey was in Dutch, the native language of all participants. Patients were recruited via 3 methods: (1) Patients participating in the Characterizing Rate of Progression in USHer Syndrome study (ClinicalTrials.gov Identifier NCT04820244) were invited to complete the survey (n = 31 invitations); (2) The otolaryngologist from the Radboud University Medical Center in Nijmegen, the National Usher Syndrome Expert center, screened the patient database on USH2a patients with a genetic diagnosis and invited them to participate in the survey (n = 51 invitations); and (3) Patient organizations spread information about the study (he survey was promoted via the website of Stichting Ushersyndroom and the magazine “Raakvlak” [Stichting OOR&OOG and Participatiegroep DoofBlinden]). In total, 75 patients that met the inclusion criteria signed up for the survey, of which 56 completed the survey, representing approximately 15% of the Dutch USH2a patient population. The control population consisted of 120 participants. Participants for the control population were recruited via social media and by asking the patients to distribute the survey to nonaffected family members. In the recruitment of control individuals, poor sleep (quality) was not an exclusion criterion. The only exclusion criterion for control participants was the presence of a visual impairment that could not be corrected by glasses. The mean age of the USH2a population was 43.34 ± 13.75 years, and the control population was 41.33 ± 14.68 years. The survey was filled out online via Castor Electronic Data Capture (https://castoredc.com). Accessibility of the questionnaire was validated by a patient panel. Patients had the possibility to complete the survey in multiple phases to prevent their answers from being affected by tiredness. All data were collected between November 2021 and January 2022.

### Measures

Five validated questionnaires were used in the survey: the Pittsburgh Sleep Quality Index (PSQI), Holland Sleep Disorders Questionnaire (HSDQ), Morningness–Eveningness Questionnaire (MEQ), Checklist Individual Strength (CIS), and Epworth Sleepiness Scale (ESS). The PSQI is designed to assess sleep quality and disturbances. It consists of 19 items, such as usual bedtime, how long it usually takes to fall asleep, and how often the participant has trouble sleeping because of 9 different situations presented (e.g., waking up in the middle of the night or feeling too hot or cold). Besides the global PSQI score, 7 component scores can be calculated: subjective sleep quality, sleep latency, sleep duration, habitual sleep efficiency, sleep disturbances, use of sleep medication, and daytime dysfunction.^18^ The HSDQ screens for 6 categories of sleep disorders: insomnia, sleep-related breathing disorder, hypersomnia, circadian rhythm sleep–wake disorder, parasomnia, and sleep-related movement disorders, such as restless legs syndrome and periodic limb movement disorder. This questionnaire consists of 32 items that could be rated on a 5-point scale ranging from “not at all” to “completely.” A total score and 6 scores for the individual sleep disorders can be calculated.^19^ The MEQ is designed to determine a person’s chronotype. It consists of 19 questions, which are mostly focusing on preferred schedule and energy levels during the day. All questions are multiple choice, with 4 to 6 answer options. The calculated score is an indicator for a participant being a definite/moderate evening or morning type or an intermediate type.^20^ The CIS is used to quantitatively assess fatigue. It consists of 20 statements that can be answered on a 7-point scale ranging from “yes, that is true” to “no, that is not true.” The participant indicates to what extent the particular statement applied to them over the past 2 weeks. Specific questions of the CIS questionnaire can also be used to calculate 4 different dimensions of fatigue: the subjective experience of fatigue, reduction in motivation, reduction in activity, and reduction in concentration.^21^^,^^22^ The ESS is used to assess daytime sleepiness. The participant rates the chance of falling asleep or dozing off on a 4-point scale for 8 different situations presented.^23^ All questionnaires were available in Dutch. In addition to these 5 questionnaires, the patients were asked whether they had a genetic diagnosis (inclusion criterion), and their year of birth. The control participants were asked whether they had a diagnosed visual impairment (exclusion criterion) and their year of birth.

### Visual Field Size and Visual Acuity

A subset of the participants also participated in the Characterizing Rate of Progression in USHer Syndrome natural history study and agreed to share their visual field (VF) and visual acuity (VA) data. All measurements were performed at a maximum of 1 year before or 6 months after filling in the sleep survey and were measured in the same center by the same optometrist. VF was measured with full-field automated static perimetry.^24^ The VF mean deviation (MD) is the average difference in VF sensitivity compared with the mean sensitivity of an age-matched normal observer. An MD between 0 and 2dB represents normal vision, whereas higher MD values represent a lower VF. For this study, the VF MD of both eyes was averaged. For VA, the letter score of the best functioning eye was used. Letter scores can range from 0 to 100, where 100 is the score for the sharpest vision. Average healthy participants score 84 to 88.

### Data Analysis

Sleep scores were calculated according to the description of the respective questionnaire with a custom-made R script. To compare the scores of the patient and control populations, a Wilcoxon rank-sum test was performed. Differences between the numbers of scores above versus below the threshold recommended by the questionnaire developers were tested with the chi-square test of independence. Distributions of the MEQ scores of multiple (sub)populations were compared with the Kolmogorov–Smirnov test. Correlations were tested with Pearson’s *r*. All statistical tests were performed in RStudio (version 1.4.1717). Graphs were made with the ggplot2 package in R.

## Results

### Prevalence and Character of Sleep Disturbances and Fatigue in USH2a Patients

#### Sleep Quality

**Figure 1:**
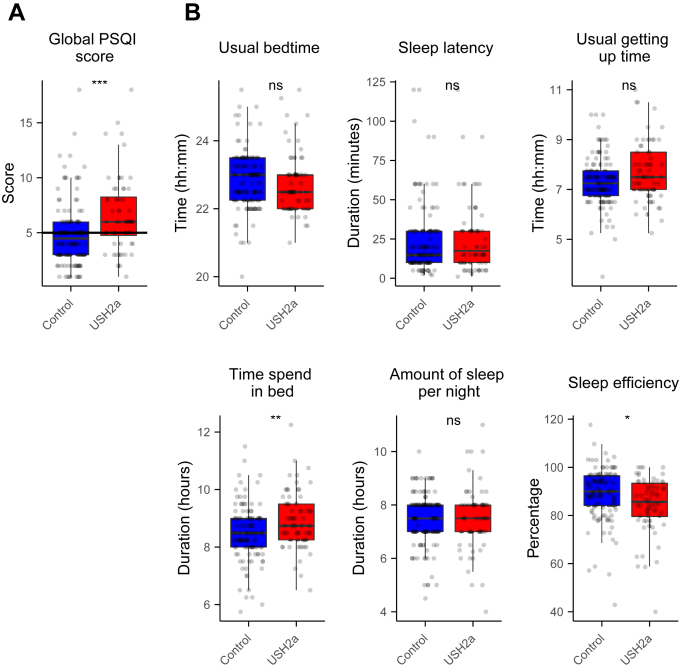
Sleep quality and efficiency based on the Pittsburgh Sleep Quality Index (PSQI) questionnaire. **A**, Global PSQI scores. The black horizontal line indicates the cutoff value for poor sleep quality. **B**, Parameters for sleep efficiency. All individual participant scores are shown as dots. Boxes show first to third quartile, in which the black horizontal line indicates the median. Whiskers extend to the most extreme data point in the 1.5 interquartile range. Scores were compared with the Wilcoxon rank-sum test (∗*P* < 0.05; ∗∗*P* < 0.01; ∗∗∗*P* < 0.001). USH = Usher syndrome.

The PSQI questionnaire was 1 of the 5 instruments in our survey to investigate sleep quality and sleep disturbances in USH2a patients. The global PSQI score of the control population was 4.903 ± 3.013 (mean ± standard deviation). The patient population scored significantly higher than the control group, with a global score of 6.768 ± 3.432 (Fig 1A; Wilcoxon rank-sum test: *P* = 1.2 × 10^−4^), indicating a lower sleep quality. Participants with a global PSQI score > 5 are classified as poor sleepers.^18^ Thirty-four of 114 (30%) of the control participants scored above this cutoff value, compared with 36 of 56 (64%) of the patients (chi-square test: 18.412; *P* = 1.8 × 10^−5^). The component scores showed that there were no significant differences in sleep duration and sleep latency. However, USH2a patients reported a significantly lower subjective sleep quality and sleep efficiency and more daytime dysfunction and sleep disturbances. In addition, sleep medication was used more often in the patient population (Table 1).

**Table 1:** Pittsburgh Sleep Quality Index Component Scores

Sleep Variable	Control Group∗	Patients with USH2a∗	*P* Value†
Global PSQI score	4.903 ± 3.013	6.768 ± 3.432	1.2e−4
Sleep duration	0.307 ± 0.680	0.518 ± 1.009	0.45
Sleep latency	1.219 ± 0.993	1.250 ± 1.083	0.94
Sleep disturbances	1.184 ± 0.452	1.446 ± 0.601	4.2e−4
Subjective sleep quality	0.947 ± 0.702	1.304 ± 0.658	1.66e−3
Daytime dysfunction	0.684 ± 0.642	1.089 ± 0.695	4.02e−4
Sleep efficiency	0.421 ± 0.739	0.714 ± 0.929	0.014
Sleep medication	0.140 ± 0.547	0.446 ± 0.971	0.013

PSQI = Pittsburgh Sleep Quality Index; USH2a = Usher syndrome type 2a.

∗ Mean ± standard deviation

† Wilcoxon rank-sum test

In Figure 1B, several parameters on sleep length and sleep efficiency are shown. The usual bedtime of the control population was 22:51 hours ± 52 minutes. The USH2a patients went to bed only a few minutes earlier, with a usual bedtime of 22:46 hours ± 55 minutes (Wilcoxon rank-sum test: *P* = 0.20). The average sleep latency of the 2 groups was almost identical, with 25.155 ± 23.26 and 26.156 ± 23.71 minutes for the control and patient cohorts, respectively (Wilcoxon rank-sum test: *P* = 0.83). Compared with the control participants, the USH2a patients got out of bed slightly later (07:20 hours ± 60 minutes and 07:43 hours ± 73 minutes, respectively; Wilcoxon rank-sum test: *P* = 0.061). Because the patients went to bed slightly earlier and got out of bed slightly later, their time spent in bed was significantly higher (control: 8.474 ± 0.971; patient: 8.945 ± 1.005; 28 minutes difference; Wilcoxon rank-sum test: *P* = 0.003). The self-reported hours of sleep per night were very comparable: 7.499 ± 1.031 for controls, and 7.5148 ± 1.210 for patients (Wilcoxon rank-sum test: *P* = 0.82). Because the time spent in bed was higher in the patient population, but the hours of sleep per night were the same, the sleep efficiency of the average patient is slightly lower than the controls (control: 88.95% ± 10.884%; patient: 84.35% ± 11.795%; Wilcoxon rank-sum test: *P* = 0.010).

**Figure 2:**
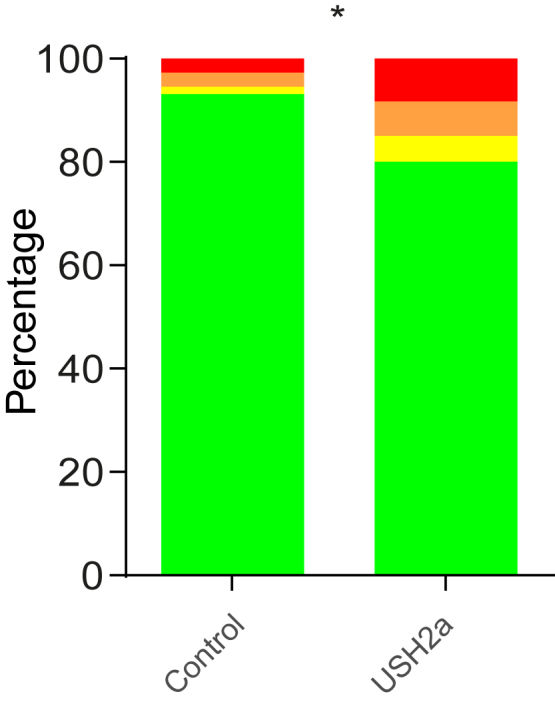
Percentage of participants using medication to help them sleep. Assessed by the Pittsburgh Sleep Quality Index question “During the past month, how often have you taken medication (prescribed or over the counter) to help you sleep?” *Green*, not during the past month; *Yellow*, less than once a week; *Orange*, once or twice a week; *Red*, ≥ 3 times a week. Number of participants with or without medication were compared with a chi-square test of homogeneity (∗*P* < 0.05). USH = Usher syndrome.

In the PSQI, the question “During the past month, how often have you taken medication (prescribed or over the counter) to help you sleep?” was asked. Seven percent of the control population used medication to help them sleep during the past month, and 2% used it ≥ 3 times a week. In the patient population, this was 20% and 9%, respectively (Fig 2; chi-square test: 6.030; *P* = 0.014). Ten patients indicated the medications they were taking, ranging from painkillers without prescription (n = 2), antihistamines (both with and without prescription; n = 2), melatonin (n = 1 prescribed; n = 2 over the counter), to antipsychotics (n = 1) and benzodiazepine receptor agonists by prescription (n = 4). The intake frequency of melatonin and benzodiazepine agonists ranged from “when necessary” to ≥ 3 times a week.

#### Sleep Disorders

**Figure 3:**
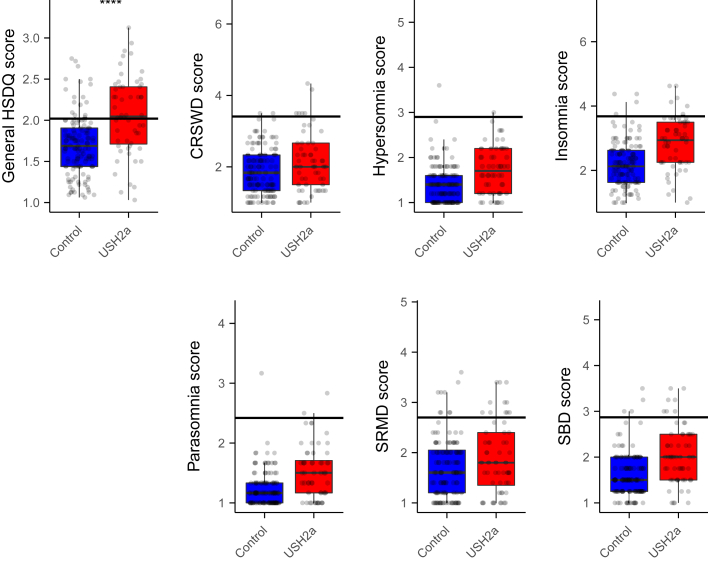
Prevalence of sleep disorders as assessed with the Holland Sleep Disorders Questionnaire (HSDQ). Black horizontal lines indicate cutoff value for sleep disorders. General HSDQ scores were compared with the Wilcoxon rank-sum test (∗∗∗∗*P* < 0.0001). CRSWD = circadian rhythm sleep–wake disorder; SBD = sleep-related breathing disorders; SRMD = sleep-related movement disorders; USH2a = Usher syndrome type 2a.

The HSDQ was used to investigate if respondents were suspected to suffer from specific sleep disorders. The control population scored 1.70 ± 0.372 for the general HSDQ score. With a general score of 2.06 ± 0.477, the patient population scored significantly higher (Fig 3; Wilcoxon rank-sum test: *P* = 1.1 × 10^−6^). Scores exceeding the cutoff value of 2.02 indicate a general sleep disorder.^19^ Seventeen of 120 (14%) of the control participants had a general HSDQ score above the cutoff, compared with 31 of 56 (55%) of the USH2a patients (chi-square test: 32.661; *P* = 1.1 × 10^−8^). Although the patients scored higher for most types of sleep disorders, the majority of the patient population scored below the cutoff values for individual sleep disorders. The prevalence of circadian rhythm sleep–wake disorder, insomnia, sleep-related movement disorder, and sleep-related breathing disorder was significantly higher in the patient population (chi-square test; Table 2), with the highest significance for circadian rhythm sleep–wake disorder. No significant differences were found in the prevalence of hypersomnia and parasomnia in the 2 populations (chi-square test; Table 2).

**Table 2:** Prevalence of sleep disorders according to the HSDQ

Sleep Variable	Control Group, %∗	Patients with USH2a, %∗	Chi-square†	*P* Value†
General HSDQ score	14	55	32.661	1.1e−08
CRSWD	1.7	11	7.204	7.3e−3
Hypersomnia	0.8	1.8	0.308	0.58
Insomnia	3.3	11	3.882	0.049
Parasomnia	0.8	3.5	1.709	0.19
SRMD (RLS/PLMD)	7.5	20	5.59	0.018
SBD	3.3	13	5.476	0.019

CRSWD = circadian rhythm sleep–wake disorder; HSDQ = Holland Sleep Disorders Questionnaire; SRMD (RLS/PLMD) = sleep-related movement disorders (restless legs syndrome/periodic limb movement disorder).

∗ Percentage of participants above the cutoff value

† Chi-square test

#### Chronotype

**Figure 4:**
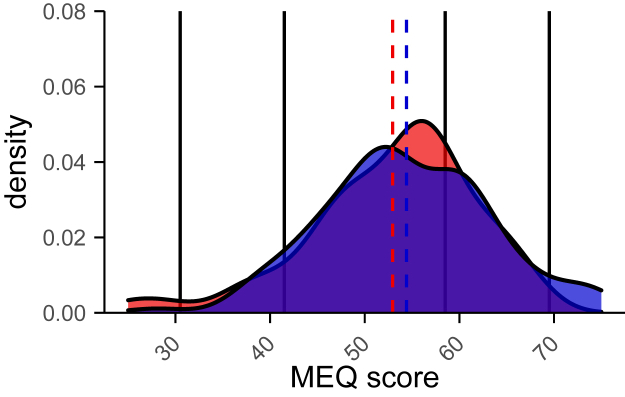
Chronotype distribution based on the Morningness–Eveningness Questionnaire (MEQ). Control population is shown in blue; patient population is shown in red. No significant difference in chronotype was found between both groups (Kolmogorov–Smirnov test; D = 0.1292; *P* = 0.55). Scores 16 to 30 indicate definite evening types, 31 to 41 indicate moderate evening types, 42 to 58 indicate intermediate chronotypes, 59 to 69 indicate moderate morning types, and 70 to 86 indicate definite morning chronotypes. Dotted lines represent the mean MEQ score of both groups.

The MEQ was used to determine the participants’ chronotype. The patient population had an MEQ score of 52.95 ± 8.94. The control population scored 54.41 ± 8.85. There was no significant difference found between the chronotype distribution of the groups (Kolmogorov–Smirnov test; D = 0.1292; *P* = 0.55). In both groups, the majority of the participants were classified as intermediate chronotypes, followed by moderate morning chronotypes (Fig 4).

#### Fatigue

**Figure 5:**
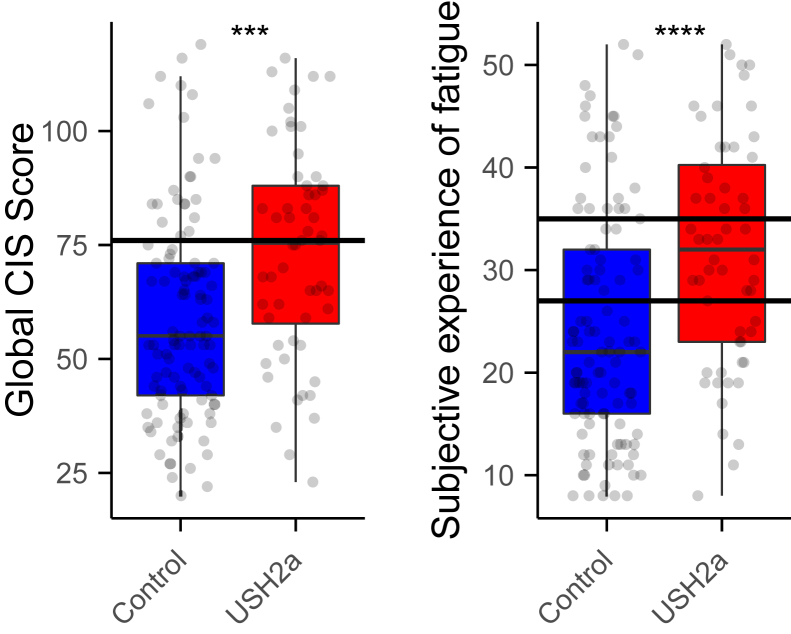
Levels of fatigue as assessed by the Checklist Individual Strength (CIS). **A,** Global CIS scores. Black horizontal line indicates the cutoff value for problematic fatigue. **B**, Scores for the dimension “subjective experience of fatigue.” Black horizontal lines indicate the cutoff values for abnormal (score > 26) or severe fatigue (score > 35). Absolute scores were compared with the Wilcoxon rank-sum test (∗∗∗*P* < 0.001; ∗∗∗∗*P* < 0.0001). USH = Usher syndrome.

Next, the CIS questionnaire was used to investigate fatigue in USH2a patients. With this questionnaire, the global CIS score and 4 scores for different dimensions of fatigue were calculated. The control population had a global CIS score of 59.24 ± 22.56, whereas the patient population scored significantly higher with 73.20 ± 23.17 (Wilcoxon rank-sum test: *P* = 3.2 × 10^−4^; Fig 5A). A global CIS score above 76 indicates problematic fatigue.^25^ Twenty-two of 105 (21%) of the control participants scored above this cutoff value, compared with 26 of 56 (46%) of the patients (chi-square test: 11.328; *P* = 7.6 × 10^−4^). Subjective experience of fatigue is 1 of the 4 dimensions that can be calculated. The control population scored 24.14 ± 11.51 compared with 31.75 ± 11.11 for the patient population (Wilcoxon rank-sum test: *P* = 7.6×10^−5^; Fig 5B). For this dimension, values above 26 indicate abnormal fatigue,^21^ which was the case in 35% of the control population and 68% of the patient population. A “subjective experience of fatigue” score > 35 indicates severe fatigue.^26^ Twenty-two of 105 (21%) of the control participants scored above the threshold indicative for severe fatigue versus 22 of 56 (39%) of the patients (chi-square test: 15.64; *P* = 4.0 × 10^−4^). In addition, patients scored significantly higher for the dimensions “Reduction in motivation” (control: 11.43 ± 5.06; patient 14.02 ± 5.53; Wilcoxon rank-sum test: *P* = 4.3 × 10^−3^) and “Reduction in activity” (control: 8.56 ± 4.50; patient: 11.45 ± 4.60; Wilcoxon rank-sum test: *P* = 1.5 × 10^−4^; Fig S6, available at www.ophthalmologyscience.org). No significant difference was found for the dimension “Reduction in concentration” (control: 15.10 ± 6.91; patient: 15.98 ± 6.13; Wilcoxon rank-sum test: *P* = 0.28). Altogether, these results show that USH2a patients suffer from fatigue more often than the control respondents.

#### Daytime Sleepiness

**Figure 7:**
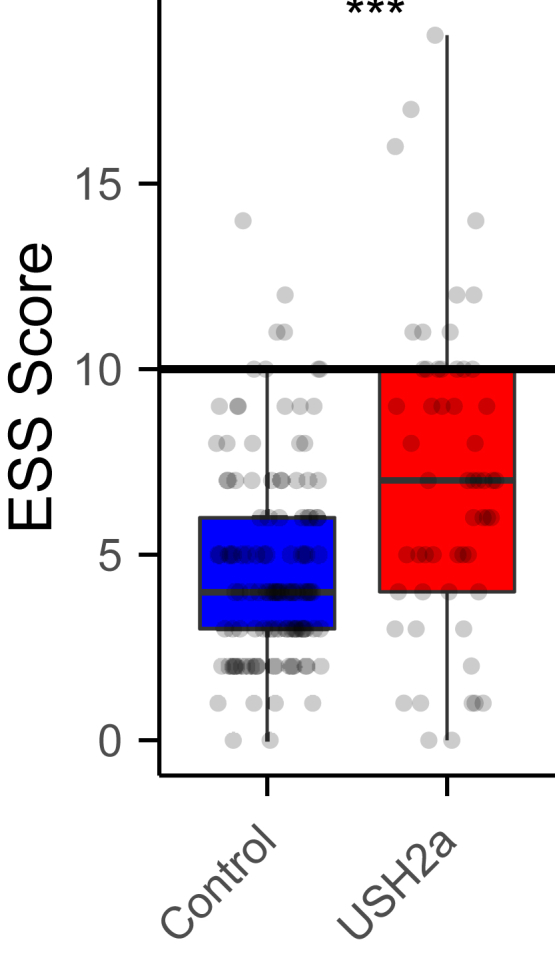
Epworth Sleepiness Scale score representing daytime sleepiness. All individual participant scores are shown as dots. The black horizontal line indicates the cutoff value for excessive daytime sleepiness. Absolute scores were compared with the Wilcoxon rank-sum test (∗∗∗*P* < 0.001). ESS = Epworth Sleepiness Scale; USH2a = Usher syndrome type 2a.

Lastly, the ESS was used to assess excessive daytime sleepiness. The ESS score of the control population was 4.725 ± 2.716, whereas the patient population scored significantly higher with a score of 7.036 ± 4.212 (Fig 7; Wilcoxon rank-sum test: *P* = 1.7 × 10^−4^). A score of ≥ 10 may indicate excessive daytime sleepiness.^23^ Only 8 of 120 (7%) of the control population had an ESS score of ≥ 10, compared with 16 of 56 (29%) of the patients (chi-square test: 15.556; *P* = 8 × 10^−5^).

### Potential Correlations

#### Daytime Sleepiness Versus Sleep Quality and Chronotype

By combining the data from all 5 questionnaires, potential correlations can be examined. For example, daytime sleepiness has been correlated with both oversleeping and too little sleep.^27^^,^^28^ Therefore, the ESS scores were used to divide the patient population into a high and low daytime sleepiness subgroup. The general sleep quality score, sleep duration, sleep efficiency, and time spent in bed data from the PSQI questionnaire of both subgroups were compared with each other. High daytime sleepiness was generally not correlated with poor sleep quality and efficiency (Fig S8, available at www.ophthalmologyscience.org). The only significant difference found was a slightly shorter time spent in bed for the patients with high daytime sleepiness (Wilcoxon rank-sum test: *P* = 7.6 × 10^−3^), but, with an average of 8.516 ± 0.636 hours and no significant difference compared with the control population (Wilcoxon rank-sum test: *P* = 0.94), this could not be described as aberrant behavior. In addition, high daytime sleepiness can be a result of an unconventional chronotype, caused by either an intrinsic disorder (e.g., delayed sleep phase syndrome) or extrinsic factors such as shift work.^29^ Therefore, the MEQ was used to examine the chronotype distribution of the patients with increased daytime sleepiness (indicated by an ESS score of ≥ 10). As shown in Figure S9 (available at www.ophthalmologyscience.org), this group had an MEQ score of 56.94 ± 6.59 (intermediate chronotype) with a second density peak approximately 67 (moderate morning type). No significant difference was found when compared with the entire patient population (Kolmogorov–Smirnov test: D = 0.2054; *P* = 0.67) or compared with the control population (Kolmogorov–Smirnov test: D = 0.2626; *P* = 0.29). This indicates that the high daytime sleepiness in these cases is not related to an urge to be awake during nighttime.

#### Fatigue Versus Sleep Quality

To examine a potential relationship between fatigue and sleep quality, the CIS scores of all patients were compared using the same 4 parameters as above. No correlation was found between the level of fatigue and the time spent in bed, sleep duration, or sleep efficiency. However, USH2a patients with a high self-reported fatigue (indicated by a CIS score > 76) generally also had a worse sleep quality, indicated by a higher PSQI score (Wilcoxon rank-sum test: *P* = 0.012; Fig S10, available at www.ophthalmologyscience.org).

#### Sleep Medication Versus Sleep Disturbances

The patients who used medication to help them sleep generally did not have lower PSQI, HSDQ, CIS, and ESS scores than the other patients (Fig S11, available at www.ophthalmologyscience.org). However, both the type of medication and intake frequency of the patients varied. In addition, the group is rather small, and it is unknown whether the sleep quality of the patients was even worse before taking sleep medication. However, the high use of medication to improve sleep can be seen as another indication of a high prevalence of sleep problems in USH2a patients.

### Sleep Disturbances and Disease Progression

**Figure 12:**
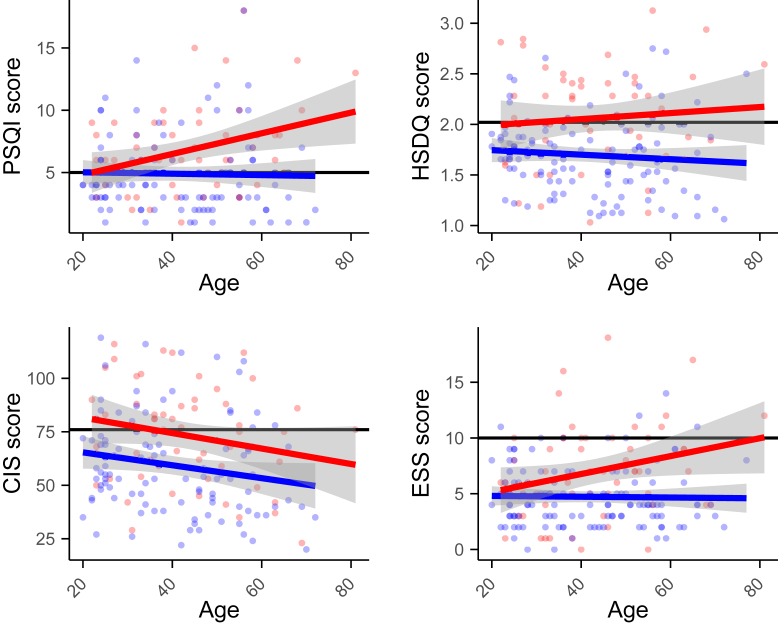
Pittsburgh Sleep Quality Index, Holland Sleep Disorder Questionnaire (HSQDQ), Checklist Individual Strength (CIS), and Epworth Sleepiness Scale (ESS) scores plotted against age. Red and blue lines represent regression lines of the patient and control population, respectively. Individual participants are shown as dots. Black horizontal lines represent the cutoff values for problematic fatigue/sleep. PSQI = Pittsburgh Sleep Quality Index.

Patient anamneses indicated that sleep problems are often experienced already long before the onset of visual impairment. To investigate whether there is a correlation between the sleep problems and the level of visual dysfunction, the global CIS, ESS, HSDQ, and PSQI scores were plotted against 3 parameters indicative of disease progression. Because USH2A*-*associated vision loss is progressive, the age of the participants was the first parameter. In Figure 12, the CIS, ESS, HSDQ, and PSQI scores of each participant were plotted against their age. As indicated by the red dots, several USH2a patients aged < 30 years reported high levels of fatigue and poor sleep quality. In general, this group still has sufficient visual function for light perception and synchronization of the circadian clock, because Pierrache et al^30^ showed that only 4% of all USH2a patients are legally blind at the age of 30 years (n = 152; Goldmann VF < 10). The correlation between age and sleep scores was tested with a linear regression analysis. No significant correlation was found between age and the following 3 sleep parameters: (1) fatigue (CIS; *P* = 0.11), (2) daytime sleepiness (ESS; *P* = 0.051), and (3) sleep disorders (HSDQ; *P* = 0.53). A significant correlation was found between age and sleep quality (PSQI; *P* = 0.012) in USH2a patients, although this correlation has low strength (*R*^*2*^ = 0.094). Altogether, this indicates that there is no relationship between age—an indicator of disease progression—and the level of fatigue, daytime sleepiness, and sleep disorders. A small correlation between age and sleep quality was seen, in that older USH2a patients generally experience a slightly lower sleep quality.

**Figure 13:**
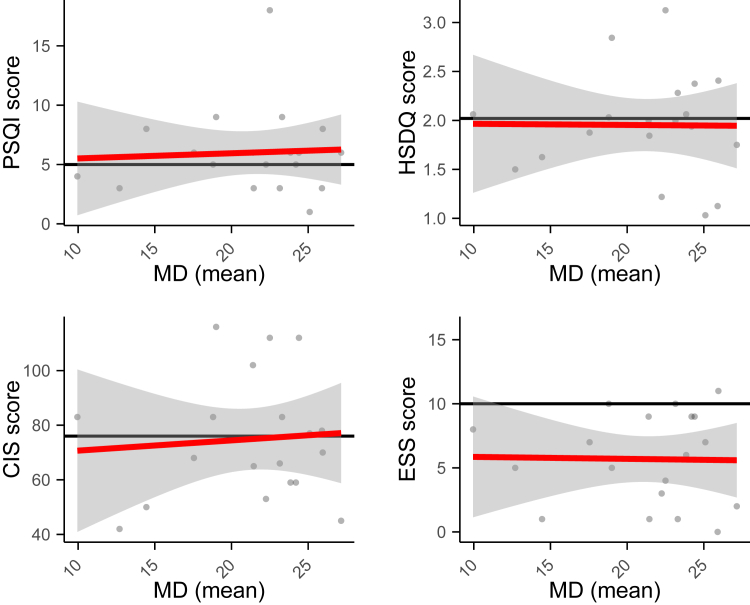
Pittsburgh Sleep Quality Index, Holland Sleep Disorder Questionnaire (HSDQ), Checklist Individual Strength (CIS), and Epworth Sleepiness Scale (ESS) scores plotted against visual field. Visual field was shown as the average mean deviation (MD) of both eyes (n = 20). Red line represents regression line. PSQI = Pittsburgh Sleep Quality Index.

Although USH2A-associated vision loss is progressive, the age of onset and level of visual dysfunction can vary.^11^^,^^30^ Therefore, age was the first and most accessible parameter for disease progression. However, VA and VF measurements would be more accurate disease progression parameters. Of the 56 patients that participated in this survey, 23 participants were able and willing to share their VA and VF data. In Figure 13, the CIS, ESS, HSDQ, and PSQI scores of the participants were plotted against their VF. The VF is expressed as the MD, where a higher score indicates a higher level of visual impairment. No significant correlation was found between the patients’ VF data and any of the 4 sleep parameters (*P* value linear regression line range, 0.75–0.97). A similar analysis was performed on the relationship between VA and sleep scores, and no strong correlations were found (Figure S14, available at www.ophthalmologyscience.org). Altogether, this indicates that there was no clear direct or indirect relationship between the level of visual impairment and the sleep problems and fatigue experienced by USH2a patients.

## Discussion

In this study, the prevalence, level, and nature of sleep problems and fatigue in USH2a patients were investigated*.* In addition, the relationship between these measures and the level of visual dysfunction was studied*.* Compared with the control population, USH2a patients scored significantly worse on all 4 studied parameters: sleep quality, sleep disorders, fatigue, and daytime sleepiness. No differences in chronotype of USH2a patients and controls were seen. Interestingly, this study showed that sleep disturbances and high levels of fatigue experienced by USH2a patients were not correlated to their level of visual impairment. These results were in accordance with the patients’ experiences that their sleep problems already existed before the onset of vision loss.

In the PSQI, the global score of the control population had an average of 4.9 and a median of 4.5. With the cutoff for poor sleep quality set at 5.0 by the creators of this questionnaire,^18^ the average control participant was already almost classified as a person experiencing poor sleep. In the original publication from 1989, the 52 control participants had a global PSQI score of 2.64 ± 1.70. It should be noted that this control population consisted of participants described as “good sleepers” based on participation in other studies.^18^ In more recent studies, a high global PSQI score is common. For example, in the study of Hayashino et al,^31^ the 1187 control participants with 0 comorbid conditions had an average score of 4.53 ± 2.25. In the study of Bøe Lunde et al,^32^ the control population without multiple sclerosis even scored 6.36 ± 4.1 (n = 108). In the study of Barrea et al,^33^ the baseline survey of participants prequarantine had a global PSQI score of 6.37 ± 3.96 (n = 121). The systematic review of Lee et al^34^ showed that, whereas most studies used the original cutoff score of 5, a cutoff value of 8 was also chosen in some studies. Based on these results, it could be argued that the PSQI cutoff value is too stringent. However, independent of a chosen cutoff, the large significant increase in global PSQI scores of USH2a patients compared with the control population remains a valuable outcome.

The importance of light as a synchronizer of circadian rhythmicity has been well-known for many decades.^35^^,^^36^ Although there are plenty of studies that recognize the high prevalence of sleep–wake disorders in the blind community^12^^,^^37^ or in patients with RP in particular,^13^^,^^38^ the relation between USH, sleep, and circadian rhythm is still underexposed. To our knowledge, this is the first study specifically addressing and quantifying sleep disturbances and disorders in USH patients. However, there have been previous studies briefly appointing similar observations. The first is the study by Dammeyer^14^ on the development and characteristics of children with USH. Although sleep was only a minor element of the study, a high prevalence of sleep problems, mainly in patients clinically diagnosed with USH1 and USH2, was observed, as well as the finding that this comorbidity occurs before complete loss of vision. Second, an independent study by Wahlqvist et al^16^ studied the physical and psychological health in USH2 patients. It was found that the level of fatigue was significantly higher in USH2 patients compared with the reference group. However, in their paper, sleep quality was not investigated, but fatigue was framed as one of the symptoms of poor psychological health. Third, in a study by Ehn et al,^15^ the relationship between work and health in persons with USH2 was reported. Sleep problems were identified in both the working group and the disability pension group, but the data were not compared with the healthy population. Last, in-depth interviews with 7 USH2 patients revealed that they generally have low energy levels and have to prioritize certain activities and take sufficient time to recover. This study did not focus on sleep, but physical and mental symptoms such as “constant fatigue” were described as common. It was stated that “Fatigue and stress were previously reported as the most prominent health problems among people with USH2,^16^ and stress and fatigue were found to be common in both actively working and nonworking people with USH2.^15^ This indicates that stress or fatigue may be related to other factors outside of working life.”^39^ Although these studies either used a small sample size, poorly differentiated between genetic etiology of USH, or did not consider the factors in disease progression, they provide additional evidence of sleep problems and fatigue in USH patients.

Although never studied before in such detail, the results obtained in this study are solely based on questionnaires. One of the risks of these types of studies is an enrichment for participants that experience the topic of investigation because they are more keen to participate to share their problems. To reduce this potential bias, it was clearly communicated that patients without sleep problems could also participate. In addition, control participants with known sleep problems were not excluded from participation to represent the general population as accurately as possible. Also, although extensively validated, questionnaires only reflect a subjective measure of sleep. However, fatigue and sleep quality are challenging to measure objectively, due to their inherent subjective nature.^40^^,^^41^ All results from this study point toward a high prevalence of sleep disturbances and fatigue in USH patients. No correlation was found between the experienced fatigue or daytime sleepiness and poor sleep quality. Most notably, the level of visual impairment of USH patients does not seem to be the explanation for the sleep problems, and the cause is still to be scrutinized. One possible cause might be poor psychological health, because mental health and sleep are known to be closely linked.^42^ However, in that case, it would be expected that young presymptomatic USH2a patients—still unaware of the consequences of their disease and not visually impaired yet—would experience fewer sleep problems, whereas the study of Dammeyer^14^ showed they do actually already suffer from poor sleep. For a potential follow-up study, it would be intriguing to study the sleep problems of presymptomatic USH2a patients in more detail. For example, the Children’s Sleep Habits Questionnaire could be distributed among the parents or caretakers of children diagnosed with USH2a who are presymptomatic for vision loss.^43^ Another way to address mental health as a confounding factor is to include an evaluation of psychological health in follow-up studies (e.g., the Hospital Anxiety and Depression Scale).^44^ Potential other confounders could be sex, body mass index, and activity or lifestyle. In addition, it should be noted that all questionnaires used in this study focused on the general or average experience of sleep during the last weeks or months. In this study, all data were collected during winter. It could be that some measures have a different outcome in summer because there are sleep parameters that are known to fluctuate seasonally.^45^^,^^46^ To rule out seasonal influences, data from the patients and control population were collected during the same period. In addition to seasonal differences, it is also possible to examine day-to-day fluctuations. To study sleep problems in even more detail and monitor day-to-day differences, the use of an actigraphy device or daily sleep diary, such as the consensus sleep diary, would be helpful.^47^ In such a study, several lifestyle parameters, such as the amount of activity, caffeine intake, and day-to-day fluctuations of bedtime could be considered. If follow-up experiments exclude psychological health and lifestyle as a cause of sleep disturbances, it would be useful to study more fundamental biological processes such as the circadian melatonin rhythm.

In this study, only USH2a patients were included to have the best stratified study population possible to be subjected to extensive sleep questionnaires. Currently, it is unknown whether patients with other (sub)types of USH also experience sleep problems. The study of Ehn et al^15^ focused on USH2, without making a distinction between USH2A, USH type 2C, and USH type 2D subtypes. Wahlqvist et al^16^^,^^17^ reported 2 studies: 1 on USH2 and 1 on USH1 (again without separating the different subtypes). The study of Dammeyer^14^ included both USH1 and USH2 patients. Usher syndrome proteins are known to interact with each other, forming complex protein networks.^1^^,^^48^ Therefore, it would be intriguing to study the prevalence of sleep problems in other types or subtypes of USH as well. In addition, it would be valuable to compare these results to the sleep phenotype of cases with nonsyndromic RP, for example, nonsyndromic RP caused by pathogenic variants in the *EYS* gene (which is the second most frequent cause of nonsyndromic RP),^49^ or nonsyndromic RP resulting from pathogenic variants in the USH2A gene.^30^ Last, as opposed to other types of USH or RP, it would be worthwhile examining a potential genotype–phenotype correlation between the type of pathogenic variants and the severity of the experienced sleep problems. Expanding this research to different USH (sub)types, different nonsyndromic types of RP, or the type of pathogenic variants could have multiple benefits and applications. It will not only contribute to the recognition of sleep problems among the broader USH or RP population but, also, to a better understanding of the biological process underlying these sleep problems.

Based on the abovementioned studies and the results of our own study, we advocate that poor sleep quality and fatigue are highly prevalent within the USH2a population, which we feel should be added as a listed comorbidity. Recognition of sleep problems as a comorbidity would be the first step toward improved patient care. The absence of a relationship between the level of visual impairment and the severity of reported sleep problems suggests a different origin of sleep problems in the USH2a population than for the general blind community. Further research should investigate whether this phenomenon also holds true for other subtypes of USH. Unraveling the potential extraretinal origin of the sleep disturbances experienced by USH2a patients will be an important next subject for future studies.
