# Supplementary material for: Evaluation of Sleep Quality and Fatigue in Patients with Usher Syndrome Type 2a
Source: Ophthalmol Sci. 2023 May 5;3(4):100323. doi: 10.1016/j.xops.2023.100323 (PMC10272497; doi:10.1016/j.xops.2023.100323)
Supplement: Figure S6 [file mmc1.pdf]

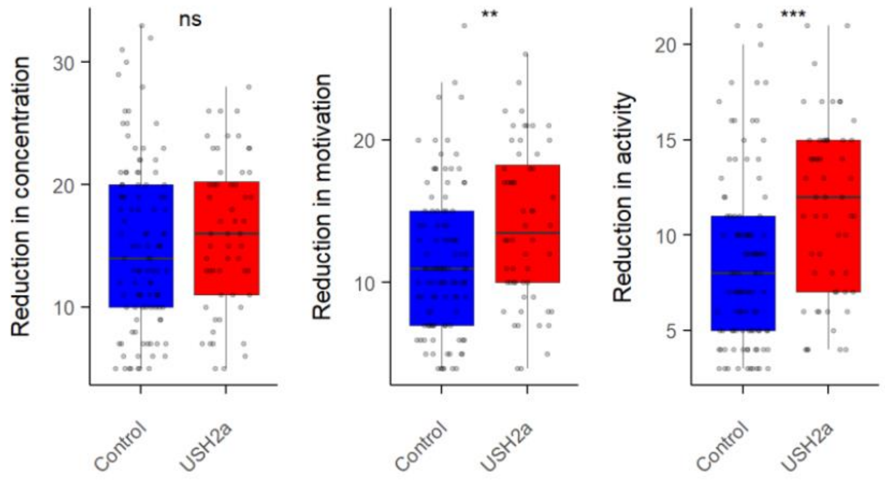


**Supplementary figure S6: CIS scores for the components reduction in concentration, motivation and activity.** Scores were compared with the Wilcoxon rank sum test (** = p < 0.01; *** = p < 0.001).
