# Supplementary material for: Evaluation of Sleep Quality and Fatigue in Patients with Usher Syndrome Type 2a
Source: Ophthalmol Sci. 2023 May 5;3(4):100323. doi: 10.1016/j.xops.2023.100323 (PMC10272497; doi:10.1016/j.xops.2023.100323)
Supplement: Figure S8 [file mmc2.pdf]

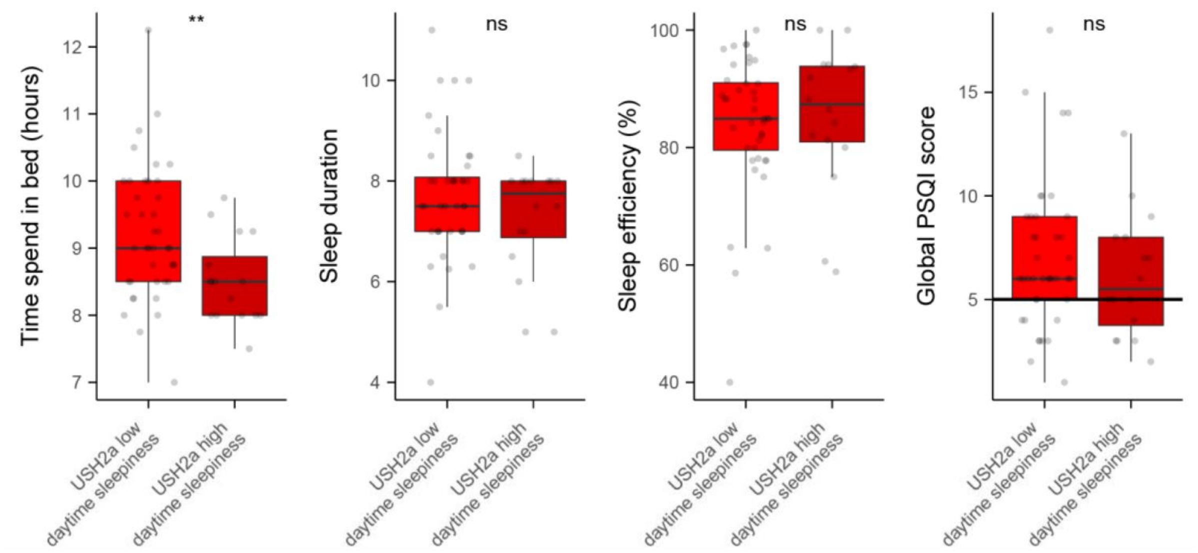

Supplementary figure S8: Time spent in bed, sleep duration, sleep efficiency, and global PSQI scores of patients with high daytime sleepiness. High daytime sleepiness was indicated by an ESS score ≥10. Scores were compared with the Wilcoxon rank sum test (** = $p < 0.01$).
