# Supplementary material for: Evaluation of Sleep Quality and Fatigue in Patients with Usher Syndrome Type 2a
Source: Ophthalmol Sci. 2023 May 5;3(4):100323. doi: 10.1016/j.xops.2023.100323 (PMC10272497; doi:10.1016/j.xops.2023.100323)
Supplement: Figure S9 [file mmc3.pdf]

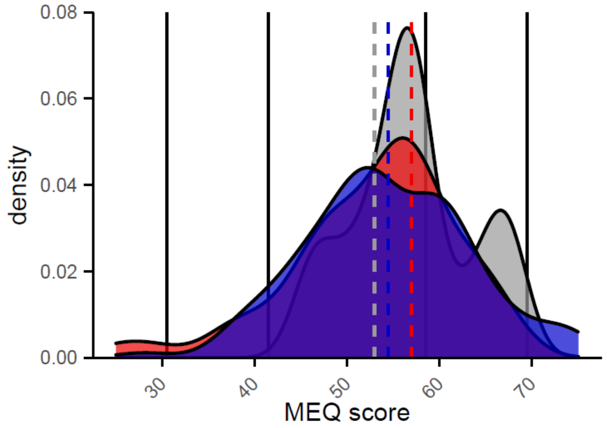

Supplementary figure S9: Chronotype distribution of patients with increased daytime sleepiness. Chronotype based on the morningness-eveningness questionnaire. Chronotype distribution of the 16 patients with increased daytime sleepiness (based on an ESS score ≥ 10) is shown in grey. Control population shown in blue, total patient population in red. No significant differences were found between the groups (Kolmogorov-Smirnov test). Scores 16-30 indicate definite evening types, 31-41 moderate evening types, 42-58 intermediate chronotypes, 59-69 moderate morning types, and 70-86 definite morning chronotypes. Dotted lines represent the mean MEQ score of each group.
