# Supplementary material for: Evaluation of Sleep Quality and Fatigue in Patients with Usher Syndrome Type 2a
Source: Ophthalmol Sci. 2023 May 5;3(4):100323. doi: 10.1016/j.xops.2023.100323 (PMC10272497; doi:10.1016/j.xops.2023.100323)
Supplement: Figure S10 [file mmc4.pdf]

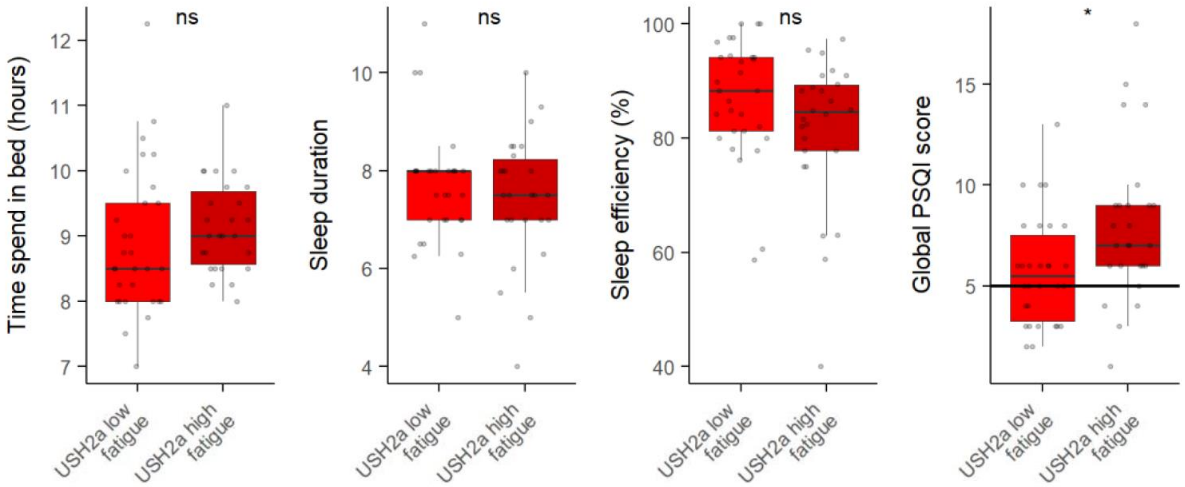

Supplementary figure S10: Time spent in bed, sleep duration, sleep efficiency, and global PSQI scores of patients with high levels of fatigue. High level of fatigue was indicated by a CIS score ≥ 76. Scores were compared with the Wilcoxon rank sum test (* = $p < 0.05$).
