# Supplementary material for: Evaluation of Sleep Quality and Fatigue in Patients with Usher Syndrome Type 2a
Source: Ophthalmol Sci. 2023 May 5;3(4):100323. doi: 10.1016/j.xops.2023.100323 (PMC10272497; doi:10.1016/j.xops.2023.100323)
Supplement: Figure S11 [file mmc5.pdf]

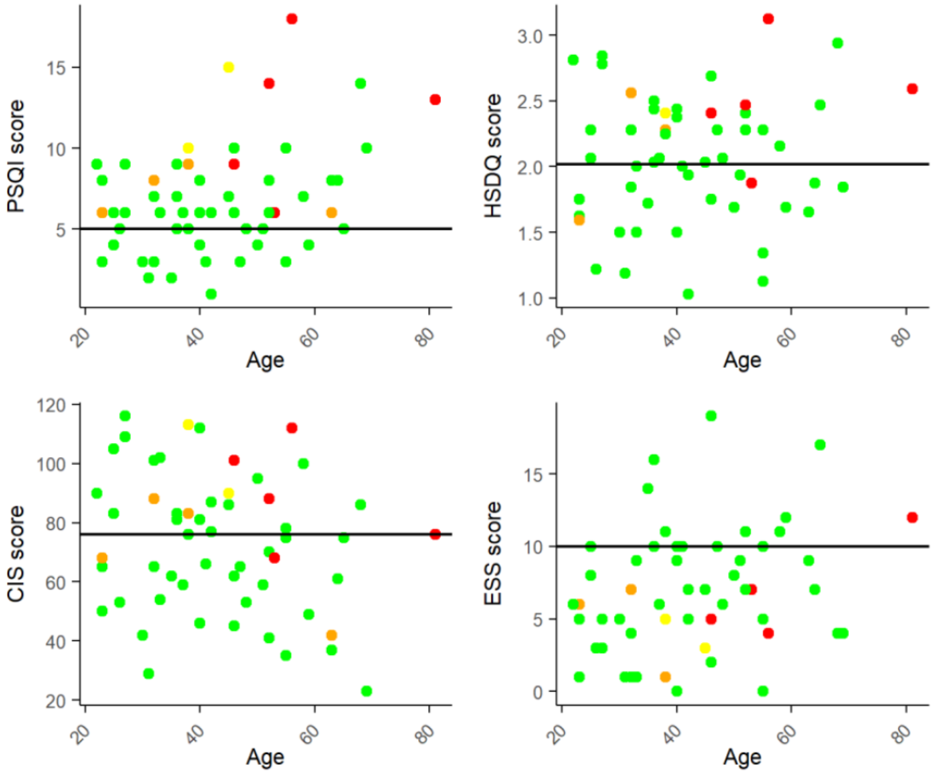

Supplementary figure S11: PSQI, HSDQ, CIS and ESS scores of all patients plotted against age. Colours of the individual dots represent the frequency of sleep medication. Green: Not during the past month; Yellow: Less than once a week; Orange: Once or twice a week; Red: Three or more times a week.
