# Supplementary material for: Evaluation of Sleep Quality and Fatigue in Patients with Usher Syndrome Type 2a
Source: Ophthalmol Sci. 2023 May 5;3(4):100323. doi: 10.1016/j.xops.2023.100323 (PMC10272497; doi:10.1016/j.xops.2023.100323)
Supplement: Figure S14 [file mmc6.pdf]

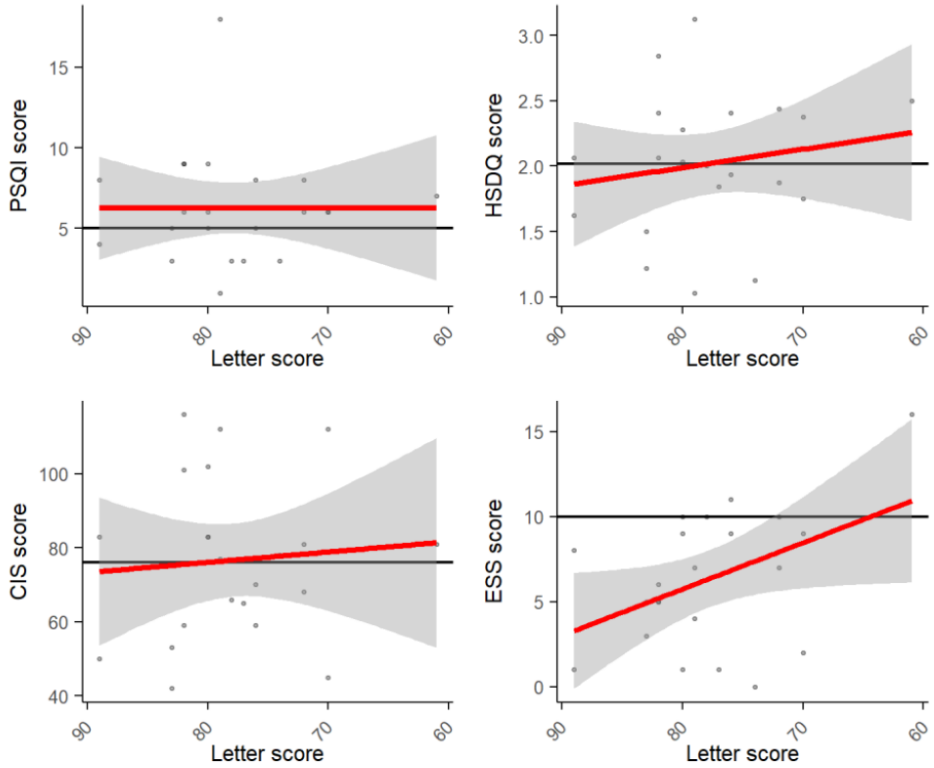

Supplementary figure S14: PSQI, HSDQ, CIS and ESS scores plotted against visual acuity. Visual acuity was shown as the letter score of the best functioning eye (n=23). Red line represents regression line. No significant correlation was found between visual acuity and PSQI, HSDQ and CIS (p-values linear regression lines 0.996, 0.44 and 0.72, respectively). A small correlation was found between visual acuity and ESS score (linear regression line p = 0.045) although this correlation has low strength (R_2 = 0.14).
